# Re‐expansion pulmonary edema after chest tube drainage of malignant pleural effusion

**DOI:** 10.1002/ccr3.6088

**Published:** 2022-08-24

**Authors:** Sachiho Inada, Hiroshi Sugimoto, Kyosuke Nakata

**Affiliations:** ^1^ Department of Respiratory Medicine Konan Medical Center Kobe Japan

**Keywords:** complication, non‐cardiac pulmonary edema, thoracentesis, thoracostomy tube

## Abstract

A 62‐year‐old man presented with a 3‐day history of dyspnea. Chest X‐ray revealed a pleural effusion. We performed chest tube drainage, and then the patient experienced re‐expansion pulmonary edema. His respiratory distress improved after the treatment of noninvasive positive pressure ventilation and intravenous methylprednisolone.

## Abstract 2

Re‐expansion pulmonary edema is an uncommon complication of chest tube drainage. Early recognition of re‐expansion pulmonary edema is critical because it is a potentially fatal complication of chest tube drainage.

## 1 CASE HISTORY

**FIGURE 1:**
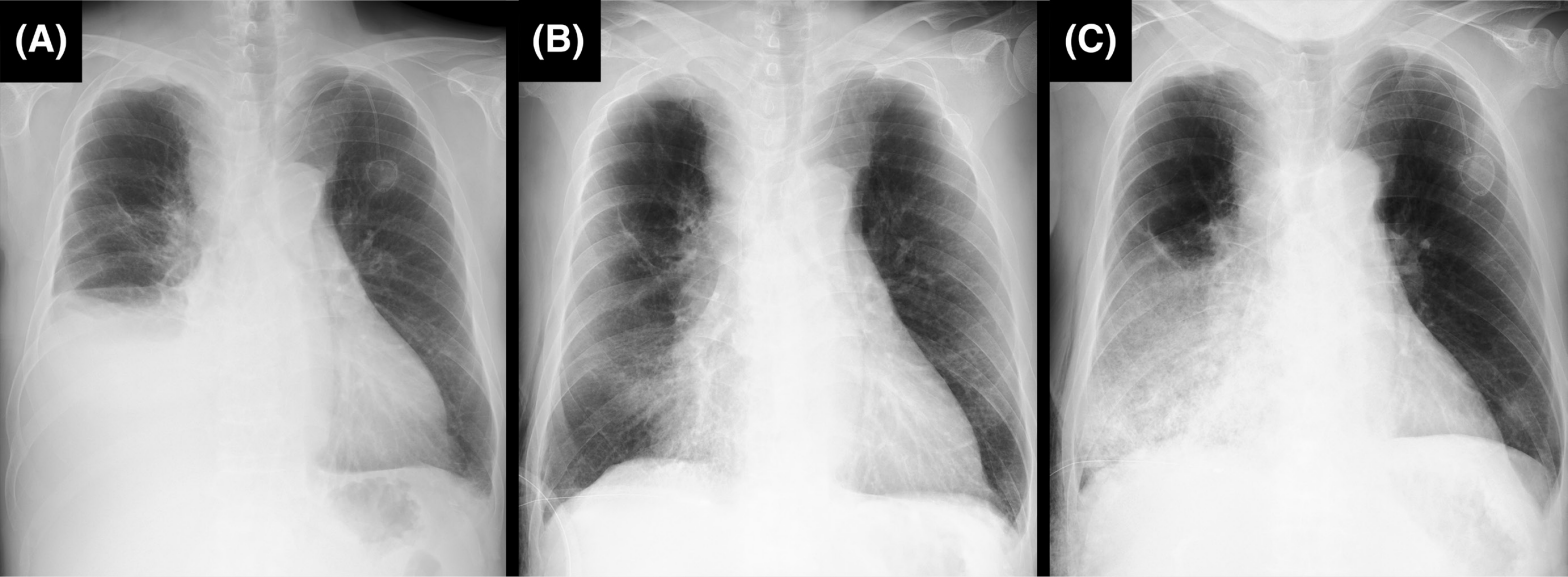
Chest X‐ray. (A) Before chest tube drainage, a pleural effusion occupied half of the right lung cavity. (B) Fifteen minutes after the insertion of the chest tube, almost all the pleural effusion disappeared. (C) Two hours after the insertion of the chest tube, ipsilateral pulmonary edema appeared

A 62‐year‐old man with metastatic lung adenocarcinoma presented with a 3‐day history of dyspnea. Chest X‐ray demonstrated a pleural effusion (Figure 1A). We performed chest tube drainage, and cytology showed adenocarcinoma. Fifteen minutes after the insertion of the chest tube, the patient experienced acute distress with coughing. Approximately 1500 ml of pleural effusion had been drained via water‐seal drainage. Chest X‐ray showed the disappearance of almost all the pleural effusion and near‐complete lung re‐expansion (Figure 1B). About 2 h later, he became acutely distressed again with a productive cough. Chest X‐ray revealed ipsilateral pulmonary edema (Figure 1C). Re‐expansion pulmonary edema (RPE) was diagnosed. He was managed with noninvasive positive pressure ventilation (NPPV) and intravenous methylprednisolone. His respiratory distress gradually improved, and he was weaned from NPPV in 4 h.

RPE is a complication of chest tube drainage, which occurs due to the re‐expansion of a collapsed lung. The incidence of RPE is higher following drainage for pneumothorax (16%) than that for pleural effusion (0.08%).^1^, ^2^ The mortality rate among patients with RPE has been reported to be as high as 25%.^2^ Early recognition of RPE is critical because it is a potentially fatal complication of chest tube drainage.

## AUTHOR CONTRIBUTIONS

SI and HS cared for the patient, collected the data, and drafted the manuscript. KN cared for the patient, collected the data, and revised the manuscript.

## CONFLICT OF INTEREST

All of the authors declare no conflicts of interest.

## CONSENT

Written consent was obtained from the patient.

## Data Availability

Data available on request from the authors.
